# Unerwartet schwieriger Atemweg bei einem Patienten mit asymptomatischen Rezidiv einer Postintubationsstenose

**DOI:** 10.1007/s00101-023-01257-5

**Published:** 2023-02-14

**Authors:** Philippe Kruse, Stefan Boskovic, Benjamin Philipp Ernst, Christian Stark, Maximilian Wetterkamp, Se-Chan Kim

**Affiliations:** 1 Klinik für Anästhesiologie und Operative Intensivmedizin, Universitätsklinikum Bonn (AöR), Venusberg-Campus 1, 53127 Bonn, Deutschland; 2 Klinik und Poliklinik für Hals-Nasen-Ohren-Heilkunde, Universitätsklinikum Bonn, Bonn, Deutschland; 3 Zentrum für Anästhesiologie, Perioperative Medizin und Schmerztherapie, RKH Orthopädische Klinik Markgröningen gGmbH, Markgröningen, Deutschland

## Anamnese

Ein 67-jähriger Patient erhielt eine Allgemeinanästhesie für eine Organtransplantation bei terminaler Niereninsuffizienz. In der anästhesiologischen Voruntersuchung zeigten sich bis auf eine therapierte Postintubationsstenose im Jahr 2012 keine weiteren relevanten Vorerkrankungen.

**Figure 1:**
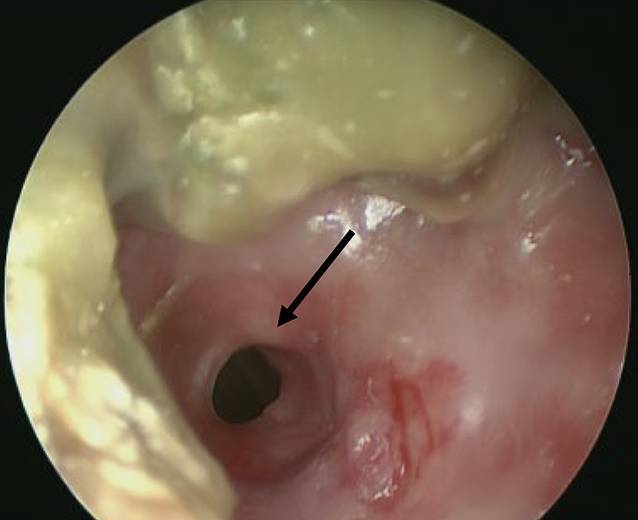

Zur Vorgeschichte der Postintubationsstenose ist anzumerken, dass diese nach einer intensivmedizinischen Behandlung bei dekompensierter Niereninsuffizienz und begleitender schwerwiegender Kreislaufinstabilität mit Intubations- und Beatmungspflichtigkeit über 13 Tage aufgetreten war. Nach der Extubation kam es zu einer respiratorischen Insuffizienz mit anschließender Reintubation und Beatmung über weitere 12 h. Im anschließenden Verlauf erhielt der Patient eine elektive Katheterimplantation für die kontinuierliche ambulante Peritonealdialyse (CAPD). Zu diesem Zeitpunkt befand sich der Patient in einem kompensierten Zustand und gab lediglich eine neu aufgetretene Heiserkeit seit 3 Tagen an. In der Videolaryngoskopie zeigte sich eine subglottische Einengung des Tracheallumens. Die Implantation des CAPD-Katheters wurde bei unsicherem Atemweg zunächst nicht durchgeführt. In der Diagnostik ergab sich eine 1 cm subglottisch liegende und 2–3 cm nach kaudal reichende glattwandige Trachealstenose. Eine Woche später wurde der Patient bei neu aufgetretener Zyanose und inspiratorischem Stridor durch den Rettungsdienst im hiesigen Notfallzentrum vorgestellt. In der Tracheobronchoskopie zeigten sich nun eine signifikante narbige Trachealstenose sowie eingetrocknete Schleimauflagerungen (Abb. 1). Diese wurde nach mikrolaserchirurgischer Trachealerweiterung durch das Einsetzen eines Tracheal-Stents Typ Dumon (14 mm Durchmesser, 40 mm Länge) (Novatech, La Ciotate, Frankreich) überbrückt.

## Untersuchung

In der präoperativen Anamnese und klinischen Untersuchung für die Nierentransplantation ergaben sich keine Hinweise auf ein Rezidiv der Stenose, weswegen sich primär für eine konventionelle oropharyngeale Intubation entschieden wurde. Der Patient konnte problemlos per Maskenbeatmung oxygeniert werden. Bei der Intubation war die Platzierung eines Endotrachealtubus der Größen 8,0–5,0 mm aufgrund einer subglottischen Enge nicht möglich. Der Patient ließ sich weiterhin komplikationslos per Maskenbeatmung ventilieren, weswegen sich im nächsten Schritt für eine Bronchoskopie über eine Larynxmaske i‑gel® der Größe 4 (Fa. Intersurgical, Sankt Augustin, Deutschland) entschieden wurde.

## Diagnostik

**Figure 2:**
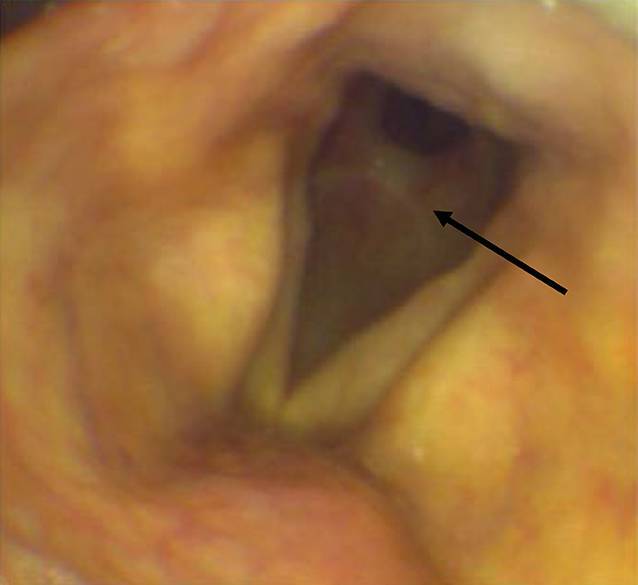

In der perioperativ durchgeführten Bronchoskopie, wofür ein Bronchoskop mit einem Durchmesser von 5,0 mm (Fa. Ambu, Bad Nauheim, Deutschland) verwendet wurde, zeigte sich 2–3 cm unterhalb der subglottischen Ebene eine bis zur Carina passierbare, ausgeprägte Stenose mit Kinking der Trachea nach posterior (Abb. 2). Aufgrund der besonderen Konfiguration der Stenose mit Kinking der Trachea war eine fiberoptische Intubation über die Stenose hinaus nicht möglich. Der hinzugerufene HNO-Konsiliarius versuchte die Intubation unter Zuhilfenahme einer starren Optik, die sich ebenfalls als frustran erwies. Zur fiberoptischen und starren Passage der Stenose wurden Endotrachealtuben der Größen 7,0 und 6,5 mm verwendet.

## Therapie und Verlauf

Im interdisziplinären Konsens wurde sich für eine chirurgische Tracheotomie entschieden, um im Sinne des mutmaßlichen Patientenwillen eine Verzögerung oder einen Abbruch der Transplantation zu verhindern. Die chirurgische Tracheotomie, die unter problemloser Beatmung mittels Larynxmaske durchgeführt wurde, sowie die anschließende Nierentransplantation verliefen primär komplikationslos. In der weiteren Diagnostik zeigte sich eine hochgradige subglottische Stenose, die mittels einer mikrolaserchirurgischen Erweiterung, Bougierung und einer Infiltration mit Triamcinolon behandelt wurde. In einer anschließenden Kontrolle zeigte sich noch eine die Subglottis zu ca. 30 % verlegende Reststenose, die durch eine erneute mikrolaserchirurgische Erweiterung mit Bougierung und Unterspritzung mit Triamcinolon weiterführend therapiert wurde. Bei zeitgerechter Wundheilung und allenfalls geringgradiger Reststenose erfolgten die problemlose Dekanülierung und nach mehrwöchigem Intervall der chirurgische Tracheostomaverschluss. Es erfolgten unauffällige laryngoskopische Kontrollen nach 3 Wochen und 3 Monaten.

## Diskussion

Pathogenetisch entsteht eine Postintubationsstenose in der Regel auf dem Boden einer Minderperfusion der Mukosa durch einen in Relation zu hohen, regionalen Druck auf die Mukosa der Trachealwand. Die konsekutive Nekrose führt schließlich zu einer narbigen Striktur [12]. Dementsprechend kann ein Cuff-Druck im Zielbereich bei einer globalen hämodynamischen Instabilität mit der Notwendigkeit einer hochdosierten Katecholamintherapie und konsekutiver Minderperfusion der Mukosa nicht mehr adäquat sein. Weitere Ursachen sind eine inadäquate Tubusgröße, lange Intubationsdauer sowie Intubation von Neugeborenen und Säuglingen in Kombination mit Infektionen [10]. Rückblickend kann nicht sicher gesagt werden, ob die systemische Hypoperfusion während der kardialen Dekompensation oder die prolongierte Intubation ursächlich ist. Hinweise auf die mutmaßliche Pathogenese hätte der gemessene Cuff-Druck während des initialen Aufenthalts auf der internistischen Intensivstation geben können. Dieser ist in der Rückschau jedoch nicht mehr nachvollziehbar. Es ist aber davon auszugehen, dass der Cuff-Druck im empfohlenen Zielbereich zwischen 20 und 30 cm H_2_O gelegen hat. Daraus folgend kann im Nachgang keine Aussage darüber getroffen werden, ob ein grenzwertig niedriger Cuff-Druck präventiv gewirkt hätte.

Das Intervall bis zum Auftreten der ersten klinischen Symptome dauert, so wie es auch bei diesem beschriebenen Fall gewesen ist, zwischen 3 bis 6 Wochen. Trachealstenosen sind auch bereits nach einer Beatmungsdauer von 24 h und nach einer Latenzzeit von 35 Jahren beschrieben worden [4, 13]. Präventiv führten die Einführung von „high-volume, low-pressure cuffs“ und die Überwachung des Cuff-Drucks zu einem Rückgang von Postintubationsstenosen.

In der hier präsentierten Kasuistik hatte der Patient bei seinem ersten Krankenhausaufenthalt über eine neu aufgetretene Heiserkeit berichtet, die sich pathophysiologisch am ehesten durch den verringerten und veränderten Luftstrom im Bereich der subglottischen Trachealstenose erklären lässt. Differenzialdiagnostisch kann eine Heiserkeit durch eine Infektion, toxische Exposition oder gastrointestinale Komplikationen ausgelöst werden. Ein weiterer anamnestischer Hinweis ist, wenn beatmete Patienten nach der Extubation aufgrund einer respiratorischen Insuffizienz unmittelbar reintubiert werden müssen [1]. Auch bei dem hier vorgestellten Patienten trat nach der Extubation eine Episode einer respiratorischen Insuffizienz mit Reintubation und kurzzeitiger Beatmungspflichtigkeit auf. Die Therapie der Trachealstenose erfolgt dabei entweder mittels Tracheaquerresektion mit End-zu-End-Anastomose der Trachea oder endotrachealer Mikrolaserchirurgie bzw. Trachealdilatation mit Stent-Einlage [3, 7]. Aktuell besteht kein interdisziplinärer Konsensus über die beste Therapiestrategie. Daher sollte bei jedem Patienten eine individuelle Therapieentscheidung in spezialisierten Zentren erfolgen [3, 11]. Eine Befundabklärung durch eine Laryngoskopie und ggf. Entnahme von Biopsien ist bei Verdacht auf Tumor, Infektion und Stimmbanddysfunktion auf jeden Fall indiziert. Das Risiko einer Restenose durch Stent-Bruch, Granulom oder überschießendes Wachstum der Mukosa ist bei der endotrachealen Therapie im Vergleich zur Tracheaquerresektion höher. Dieses war auch bei dem hier vorgestellten Patienten der Fall. Jedoch ist die Tracheaquerresektion mit einer bedeutend höheren Invasivität und in der Folge auch einem höheren perioperativen Komplikations- und Mortalitätsrisiko assoziiert [2, 8]. Im Rückblick lässt sich keine Aussage mehr darüber treffen, ob der Patient von einer chirurgischen Resektion mit anschließender Rekonstruktion, insbesondere vor dem Hintergrund der langjährigen Beschwerdefreiheit nach mikrolaserchirurgischer Trachealerweiterung und Stent-Einlage, mehr profitiert hätte.

Die „Leitlinie Atemwegsmanagement“ der Deutschen Gesellschaft für Anästhesiologie und Intensivmedizin (DGAI) und die Handlungsempfehlungen der „Canadian Airway Focus Group“ dienen als evidenzbasierte Entscheidungshilfen bei Patienten mit schwierigem Atemweg [5, 6, 9]. Für die Beurteilung der Intubationsanatomie stellt die Videolupenlaryngoskopie mittels flexibler nasaler Endoskopie ein geeignetes diagnostisches Verfahren dar. Dieses Diagnostikverfahren wird wahrscheinlich in die überarbeitete S1-Leitlinie „Atemwegsmanagement“ aufgenommen (mündliche Kommunikation). Rückblickend hätte der Patient von einer vorherigen Beurteilung der Intubationsanatomie profitiert. Daher ist zu überprüfen, ob auch bei asymptomatischen Patienten, die diese oder eine vergleichbare Historie vorweisen, zukünftig einer regelhaften Diagnostik zu zuführen sind.

## Fazit für die Praxis

Patienten nach Langzeitbeatmung oder Reintubationspflichtigkeit sollten bei neu aufgetretenem Stridor oder Heiserkeit einer Diagnostik zum Ausschluss einer relevanten Trachealstenose zugeführt werden, da diese bei Progress oder erneuter Intubation zu einem akuten Atemwegsproblem führen kann. Weiterführend stellt die Videolupenlaryngoskopie mittels flexibler nasaler Endoskopie bei Patienten mit dieser Historie, aber auch bei Patienten mit einem zu erwartenden schwierigen Atemweg anderer Genese, ein Verfahren zur Beurteilung der Intubationsanatomie dar. Voraussichtlich wird dieses in die überarbeitete S1-Leitlinie „Atemwegsmanagement“ aufgenommen (mündliche Kommunikation). Neben dieser Leitlinie kann die Handlungsempfehlung der „Canadian Airway Focus Group“ bei der Erarbeitung einer klinikinternen SOP „Atemwegsmanagement“ helfen.
